# Evidence for a Hepatitis B Virus Short RNA Fragment Directly Targeting the Cellular RRM2 Gene

**DOI:** 10.3390/cells11142248

**Published:** 2022-07-20

**Authors:** Karin Broennimann, Inna Ricardo-Lax, Julia Adler, Yosef Shaul

**Affiliations:** 1 Department of Molecular Genetics, Weizmann Institute of Science, Rehovot 76100, Israel; karin.broennimann@weizmann.ac.il (K.B.); iricardola@mail.rockefeller.edu (I.R.-L.); julia.adler@weizmann.ac.il (J.A.); 2 Laboratory of Virology and Infectious Disease, Rockefeller University, New York, NY 10065, USA

**Keywords:** deoxynucleotides and DNA viruses, RNR-R2 regulation, non-coding RNA, hepatitis B virus

## Abstract

The hepatitis B virus (HBV) is one of the smallest but most highly infectious human pathogens. With a DNA genome of only 3.2 kb and only four genes, HBV successfully completes its life cycle by using intricate processes to hijack the host machinery. HBV infects non-dividing liver cells in which dNTPs are limited. As a DNA virus, HBV requires dNTPs for its replication. HBV induces the ATR-mediated cellular DNA damage response pathway to overcome this constraint. This pathway upregulates R2 (RRM2) expression in generating an active RNR holoenzyme catalyzing de novo dNTP synthesis. Previously we reported that ERE, a small RNA fragment within the HBx ORF, is sufficient to induce R2 upregulation. Interestingly, there is high sequence similarity between ERE and a region within the R2 5′UTR that we named R2-box. Here, we established a mutant cell line in the R2-box region of the R2 gene using CRISPR-Cas9 technology to investigate the R2 regulation by ERE. This cell line expresses a much lower R2 level than the parental cell line. Interestingly, the HBV infection and life cycle were severely impaired. These cells became permissive to HBV infection upon ectopically R2 expression. These results validate the requirement of the R2 gene expression for HBV replication. Remarkably, the R2-box mutated cells became ERE refractory, suggesting that the homology region between ERE and R2 gene is critical for ERE-mediated R2 upregulation. Thus, along with the induction of the ATR pathway of the DNA damage response, ERE might also directly target the R2 gene via the R2-box.

## 1. Introduction

Hepatitis B virus (HBV) is one of the smallest DNA viruses infecting humans. Despite the available effective vaccines, there still is no cure. The virus poses a major global health threat, with almost 300 million people living with chronic HBV infections and nearly one million deaths yearly due to complications such as liver cirrhosis and hepatocellular carcinoma (WHO). HBV infects via the hepatocytes NTCP receptor, also known as SLC10A1 [1]. The virus consists of an outer membrane and a capsid contained with partially double-stranded DNA, named relaxed circular DNA (rcDNA) [2,3]. The rcDNA enters the nucleus, and the partial plus-strand DNA is elongated to completion by exploiting the cellular DNA damage repair machinery [4]. The viral DNA targets the nucleus as episomal covalently closed circular DNA (cccDNA) and is active in the transcription of the viral genes.

Because of the small size of the virus, the HBV genome is compactly organized, and the four open reading frames (ORFs) overlap each other. Several viral transcripts and transcription start sites (TSS) terminate at one common polyadenylation site. These transcripts are translated to the viral polymerase (Pol) with reverse transcriptase function, the small, middle, and large surface antigens (HBs), the Core protein that forms the nucleocapsid, and the regulatory HBx protein (Supplementary Figure S1). In addition, the long transcript, the pre-genomic RNA (pgRNA), is the template for future viral genome synthesis by the virus-encoded reverse transcriptase polymerase.

HBV infects non-dividing hepatocytes. The low deoxynucleotides (dNTPs) level in the non-dividing cells reduces the HBV DNA synthesis rate. The key enzyme in de novo dNTP synthesis is the Ribonucleotide Reductase (RNR) [5]. The RNR holoenzyme is a tetramer consisting of two R1 and two R2 subunits. R1 is continuously expressed throughout the cell cycle, whereas R2, expressed from the RRM2 gene, is restricted to the S-phase [6,7].

Previously we and others found that R2 is upregulated in the presence of HBV [8,9,10]. R2 expression and dNTP production are essential for HBV replication in non-dividing hepatocytes [8]. RNR inhibition by hydroxyurea (HU) impairs HBV progeny formation [8,11,12,13]. We also have reported that the ATR-Chk1-E2F1 DNA damage response (DDR) pathway is activated by the HBx ORF and that this process is essential for the R2 upregulation [13,14]. Lately, we reported that the integrity of the HBx ORF is dispensable for R2 upregulation. A small RNA region within the ORF, named ERE (Supplementary Figure S1), is sufficient in eliciting the ATR-Chk1-E2F1 pathway and R2 upregulation [14]. However, the questions of how ERE induces this pathway and whether this pathway is sufficient for R2 upregulation remained open. Here we report the identification of a sequence within the R2 gene that is critical for ERE to upregulate R2 expression. Remarkably, this R2 sequence is highly similar to the ERE sequence. We propose here that along with the induction of the DDR, ERE might also directly target the R2 gene for maximal R2 upregulation.

## 2. Materials and Methods

### 2.1. Tissue Culture, Treatments and Reagents

HepG2, HepG2.2.15, and HEK293T (ATCC, American Type Culture Collection) cells were cultured in Dulbecco’s modified Eagle’s medium (Gibco, Life Technologies, Thermo Scientific, Waltham, MA, USA) supplemented with 8% fetal bovine serum (Gibco) and 100 U/mL of penicillin and 100 µg/mL of streptomycin (Biological Industries, Kibbutz Beit Haemek, Israel). To obtain quiescent HepG2, the medium was supplemented with 2% dimethyl sulfoxide (DMSO, SIGMA, Welwyn Garden City, UK) for at least six days. See Supplementary Information for the list of the plasmids, primers’ sequences and list of antibodies.

### 2.2. CRISPR and Analysis of Editing

The SpCas9/sgRNA expression plasmid was described in [15] (Addgene plasmids #135012). The SgRNAs were designed using the Desktop Genetics design tool (deskgen.com, accessed on 15 April 2018). HepG2 cells were transfected with The SpCas9/sgRNA plasmid with Lipofectamine 3000 reagent (Invitrogen, ThermoFisher, Waltham, MA, USA) according to the manufacturer’s instructions. The cells were sorted after three days by a SORP-FACSAriaII for mCherry positive cells. We analyzed the genomic DNA of the edited cells as described in [16] with the Synthego ICE tool 2019. v2.0 (https://ice.synthego.com) (accessed on accessed on 15 April 2018) tool.

### 2.3. Preparation of Lentiviral Transducing Particles and Transduction

A list of the plasmids used is included in the Supplementary Information, and these include the plasmids previously described [17,18]. Lentiviruses were produced as described [8], using the calcium phosphate method to transfect HEK293T. Lentivirion-containing medium was filtered through a 0.45 µM cellulose acetate filter and supplemented with 8 µg/mL of polybrene. Virion-containing medium was used to transduce the DMSO-treated HepG2 cells for over six days. Then, 12–24 h after infection, the cells were washed five times in phosphate-buffered saline (PBS), and fresh medium was added to the cells. The transduced cells were harvested after 72 h.

### 2.4. Preparation of HBV and Lentiviral Transducing Particles and Transduction

HBV was produced from HepG2.2.15 cells as previously described [8].

HepG2 2.2.15 cells were grown with 2.5% DMSO for 1 week, and then the medium was changed to a fresh one containing 2.5% DMSO and 1% FBS. The medium was filtered with a 0.45 µm cellulose acetate filter and ultracentrifuged in a Beckham Coulter ultracentrifuge at 270,000× *g* for 16 h at 4 °C. The pellet was resuspended in TNE buffer (40 mM TRIS pH 7.5, 150 mM NaCl, 1 mM EDTA).

### 2.5. RNA and Protein Extraction and Analysis

The RNA was extracted using TRI Reagent (MRC). First-strand synthesis was performed using a qScript cDNA synthesis kit (Quantabio, Beverly, MA, USA). qRT-PCR was performed using the Light-Cycler480 (Roche) or the QuantStudio™ 12K Flex (Applied Biosystems) with PerfeCta^®^ SYBR Green FastMix mix (Quantabio). All of the qPCRs were normalized to 18S rRNA levels. The primer sequences are in the Supplementary Materials.

HBV qPCR was additionally performed using a TaqMan Universal PCR Master Mix (Applied Biosystems, Foster City, CA, USA). HBV copies/samples were calculated based on a standard curve composed from 2xHBV plasmid in a concentration range of 10^8^–10^9^ copies.

Lysates were prepared from cells using RIPA buffer [19] supplemented with Dithiothreitol (DTT) and protease and phosphatase inhibitors (Sigma) and subjected to SDS-PAGE. The antibodies are listed in the supplements. We used enhanced chemiluminescence (ECL) detection using EZ-ECL (Biological Industries).

### 2.6. Statistical Analysis

Th error bars refer to the standard error of the mean (SEM). A two-sided Student’s *t*-test was performed to assess significance.

## 3. Results

### 3.1. The Critical ERE Sequence Harbors Homology to R2 5′UTR 

**Figure 1:**
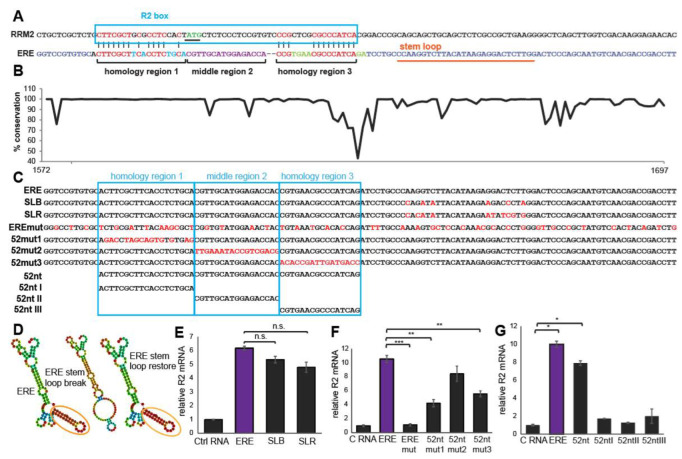
ERE sequence analysis. (**A**) The R2 sequence around the translation start codon was compared to the ERE sequence. Thirteen nucleotides leading up to the translation start site and 12 inside the coding region are homologous to the R2 transcript. We termed the homology region in the R2 locus R2-box. (**B**) Conservation of the ERE sequence among HBV genomes. Percent sequence identity per position is shown, according to ClustalW, using all HBV genomes available in the HBVDB database [20]. (**C**) ERE and mutated sequences are depicted. These mutations were cloned into lentivectors, and all the sequences contain the endogenous HBV 3′UTR to compare viral RNA expression. Abbreviations: SLB: stem-loop break: SLR: stem-loop restore; ERE mut: ERE mutated; 52mut1-3: ERE 52nt mut 1–3; ERE 52nt I–III (**D**) Structure predictions of ERE, stem-loop break and stem-loop restore mutations with RNA fold web server [21] are shown, the location of the stem-loop is indicated in panel (**A**) in orange. (**E**–**G**) Quiescent HepG2 cells were transduced with lentivectors with the respective sequence depicted in D and a negative control RNA. Cells were harvested three days after transduction, and the R2 mRNA of three biological replicates was analyzed by qRT-PCR. Student’s *t*-test was performed. n.s. = not significant, * *p*-value < 0.05, ** *p*-value < 0.01, *** *p*-value < 0.001.

We have previously reported that ERE is the HBV RNA region upregulates R2 expression 18. Sequence inspection revealed that a region within the ERE is highly homologous to the R2 5′UTR (Figure 1A). We termed the homology region in the R2 locus R2-box. To investigate the importance of the ERE region sharing homology with the R2-box, we conducted experiments to delineate the critical ERE sequence. To this end, we created a set of lentiviral constructs expressing ERE mutants and examined them for their activity to upregulate R2 expression, as previously described [14]. The ERE sequence is highly conserved among all HBV genomes available in the HBVDB database (Figure 1B) [20]. AN examination of the ERE sequence using the RNA fold webserver revealed that it is predicted to contain a stem-loop structure (Figure 1C,D) [21]. Interestingly, the stem-loop structure is potentially formed even in the context of the HBV pgRNA, the HBx ORF, and the lentiviral ERE (Supplementary Figure S2A). We changed the predicted stem sequence to impair stem-loop folding and found it as active as the wild-type (Figure 1E and Supplementary Figure S2B). Furthermore, additional mutations to restore the stem-loop structure did not obtain a gain of function results. These data suggest that the stem-loop structure is not essential for the ERE activity.

We next generated a saturated silent ERE mutant that keeps the partial HBx ORF intact. This ERE mutant was well expressed (Supplementary Figure S2C) but inactive in upregulating the RNR-R2 expression (Figure 1F, ERE mutated). We created different ERE mutants in the R2 homology region. We divided the 52-nucleotide homology region into three sub-regions, and each was mutated (Mut 1–3) and expressed in the context of the intact ERE or in isolation. The mutants in the upstream and downstream homology regions were severely impaired in R2 upregulation (Figure 1F and Supplementary Figure S2C). The 52-nucleotides long fragment, including the two homology regions, was nearly active as the full-length ERE, while the sub-regions in isolation were inactive on R2 upregulation (Figure 1G and Supplementary Figure S2D). These data suggest that the elements within ERE sharing homology with R2 are essential for HBV -mediated R2 upregulation.

### 3.2. R2-Box Is Essential for Maximal R2 Expression

**Figure 2:**
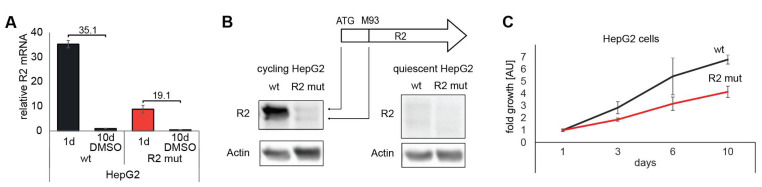
CRISPR-Cas9-targeting of RRM2 in HepG2. (**A**) Proliferating and quiescent (10 days DMSO treatment) HepG2 and HepG2 R2 mut cells were harvested, and qRT-PCR with R2 specific primers was performed. (**B**) WB analysis of R2 levels in proliferating and quiescent HepG2 and HepG2 R2 mut cells. There are two protein products for R2: a large isoform of 389 amino acids (44.9 kDa) and a small isoform of 297 amino acids (34.6 kDa). (**C**) XTT growth analysis of HepG2 and HepG2 R2 mut cells.

RNR is an essential enzyme for proliferating cells. RNR is active at the entry of the S-phase in dividing cells but barely active in the non-cycling cells. This is because of the lack of expression of the R2 (RRM2) subunit of RNR in non-cycling cells 10. We wondered whether the R2-box is important for R2 expression. To address the question, we used CRISPR-Cas9 technology to introduce deletion mutations at the 5′ UTR region, including the ATG (R2-box) in HepG2 cells (Supplementary Figure S3). This cell line expresses very low levels of R2 transcript (Figure 2A) and protein (Figure 2B). In addition to the canonical isoform, a shorter protein is likely to be initiated from the downstream ATG at position M93. The low level of R2 is enough for the cells to grow, albeit slower than naïve HepG2 (Figure 2C). The R2-box mutant cells responded to DMSO treatment to induce a quiescent state by further lowering the R2 RNA and protein level. We named the cell line HepG2 R2 mutant (R2-mut in short). These results suggest that the R2-box, the region within the R2 transcript similar to the ERE sequence, is essential for R2 expression.

### 3.3. R2 Regulates the HBV Life-Cycle

**Figure 3:**
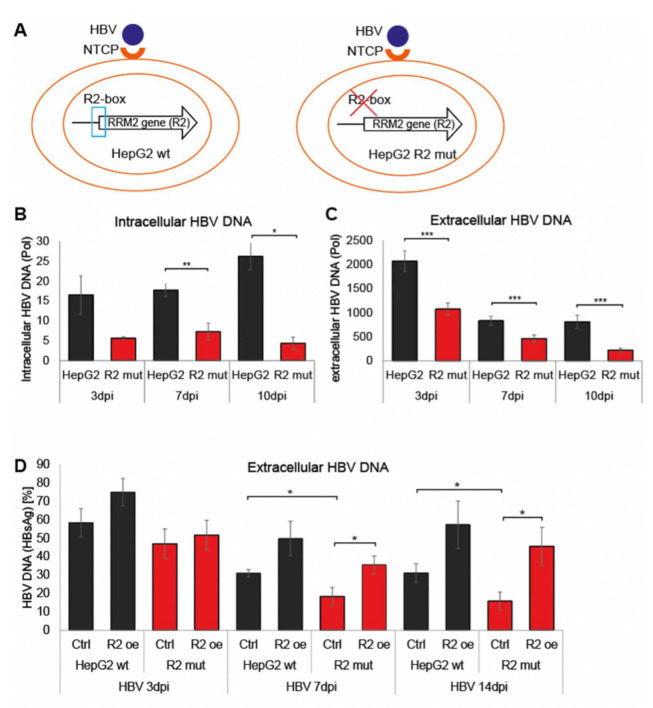
HBV proliferation is impaired in HepG2 R2 mut cell line. (**A**) schematic depiction of the employed HepG2 cells; naïve cells in left and the R2 mutant cells on the right. (**B**,**C**) Quiescent HepG2 and R2 mut cells expressing the NTCP receptor were infected with HBV (10 Genome Equivalents (GEq) per cell). Medium and cells were harvested on the indicated days post-infection (dpi). DNA was extracted and qRT-PCR with HBV-specific primers and probe was performed. The intracellular data were obtained from two different experiments, the extracellular from three experiments. (**D**) Experiment described in (**B**,**C**) was performed with R2 overexpressing cell lines (R2 oe) and qRT-PCR of extracellular HBV DNA were performed at the region of HBsAg gene. Data were taken from three independent experiments. Student’s *t*-test was performed. *p*-values: * < 0.05, ** < 0.01, *** < 0.001. dpi = days post-infection.

Next, we asked whether the HepG2 R2 mutant cells, poorly expressing R2, support HBV replication. We established naïve HepG2 and HepG2 R2-mut cell lines expressing NTCP, the HBV receptor (Figure 3A and Supplementary Figure S4A), to investigate HBV infection and expression. We infected the cells with HBV prepared from HBV-producing HepG2.215 cells, washed the inoculum away, grew the cells, and quantified HBV DNA and RNA levels over time (Supplementary Figure S4B). The naïve HepG2-NTCP were more permissive to HBV infection, as monitored by the level of HBV DNA in the cells (Figure 3B) and the medium (Figure 3C) over time (dpi = days post-infection). The amount of intra and extracellular HBV DNA was consistently significantly higher in the naïve HapG2 cells compared to the R2-mut cells. These results show that the R2 level correlates with HBV infection and propagation.

To further validate the requirement of R2 level for efficient HBV infection, we established HepG2 and HepG2 R2 mut cell lines ectopically expressing R2 (R2oe) under a CMV promoter. We quantified both the level of endogenous and ectopic R2 expression. The endogenous R2 level was not affected (Supplementary Figure S4C), but high R2 expression was obtained by the ectopically-transfected R2 construct (Supplementary Figure S4D,E). The R2 overexpression in the NTCP-HepG2 mut cells rescues HBV proliferation to levels comparable to naïve HepG2 (Figure 3D and Supplementary Figure S4F). These findings underline the importance of R2 expression for HBV replication.

### 3.4. The R2-Box Is Important for ERE-Mediated R2 Upregulation

**Figure 4:**
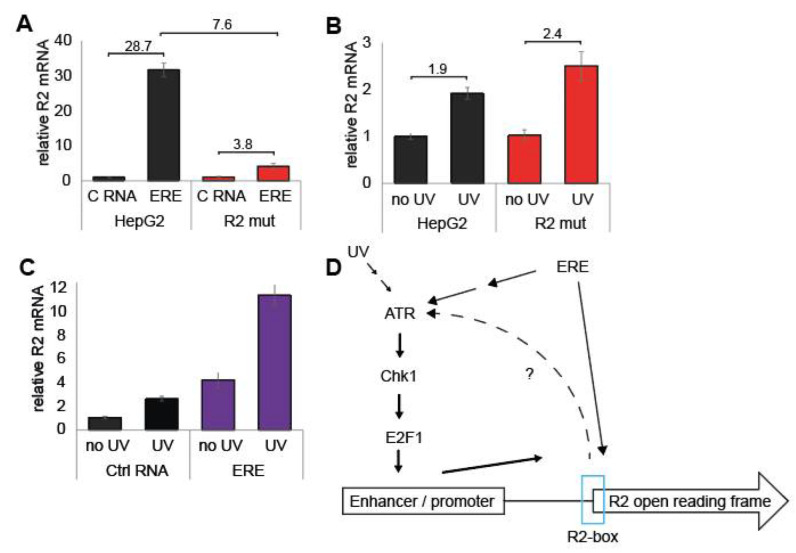
HepG2 R2 mut cells are poorly ERE responsive. (**A**) Quiescent HepG2 and R2 mut cells were transduced with ERE or control RNA. Three days after infection cells were harvested and R2 expression was measured by qRT-PCR. (**B**) Quiescent HepG2 and HepG2 R2-mut cells were UV irradiated and R2 expression was measured after 24 h. (**C**) ERE and UV have a synergistic effect on R2 upregulation. Quiescent HepG2 cells were transduced with ERE or control RNA. After three days cells were irradiated with 10 mJ/cm^2^ UV of 254 nm in a Stratalinker UV Crosslinker. After 10 h cells were harvested and RNA extracted and R2 mRNA measured by qRT-PCR. (**D**) a model describing the possible mechanisms of ERE function in upregulating R2 expression.

We have so far demonstrated that there is sequence similarity between HBV ERE and the endogenous R2 gene that we named R2-box. We have also shown that the R2-box integrity is essential for R2 expression. We, therefore, asked whether R2-box mediates ERE function. To this end, we used the HepG2 R2 mutant cells to test ERE activity. We transduced HepG2 and the R2-mut cells with ERE or the control RNA and measured R2 mRNA levels. We found that ERE upregulated R2 expression only about fourfold in the R2 mut cells, 7.6-fold less than what was obtained in the naïve HepG2 (Figure 4A). These results suggest that the ERE function of upregulating R2 is mainly mediated via the R2-box region of the endogenous R2 gene.

R2 expression is upregulated by UV [22]. UV, similar to ERE, activates the ATR-Chk1 pathway. We asked whether the R2 mutant is refractory to UV such as with ERE. Unexpectedly we found that the R2 mutants were as responsive as the naïve HepG2 to UV (Figure 4B). Furthermore, quiescent HepG2 R2-mut cells expressing ERE that were UV irradiated show a synergistic effect on R2 upregulation (Figure 4C). These data suggest that ERE is endowed with double arm functions; it activates the ATR pathway and also targets the R2-box (Figure 4D).

## 4. Discussions

HBV consumes dNTPs for its genome replication. However, dNTPs levels are too low in the quiescent hepatocytes to permit optimal HBV DNA synthesis. This is also the case with HIV. HIV DNA synthesis is inefficient in quiescent peripheral blood lymphocytes (PBLs) as compared to the activated PBLs [23]. HBV overcomes this constraint by upregulating the R2 (RRM2) gene expression, a critical component of the RNR [8,9,10], an essential enzyme in dNTP synthesis. The critical role of the dNTP level for HBV replication is exploited to fight the virus by either nucleos(t)ide analogue treatment [24] or by inhibiting the RNR enzyme [11].

Previously we reported that upon HBV infection, the ATR-Chk1-E2F1 DNA damage response pathway is activated, leading to increased R2 transcription and dNTP production [13,14]. An unexpected finding was that an embedded regulatory element (ERE) within the HBx mRNA is sufficient to induce the ATR-Chk1-E2F1 axis and upregulate the cellular R2 gene independent of protein expression [14]. Even though this is a remarkable hidden function of the HBV RNA, we could not explain the mechanism of action. Here we analyzed the ERE sequence more deeply. The functions of non-coding RNAs are usually determined by their secondary structure [25,26]. We predicted a stem-loop inside the highly conserved ERE sequence that did not end up being crucial for R2 activation. We could not determine a particular secondary structure of ERE to be involved in the mechanism of R2 upregulation.

While we characterized the 125-nucleotides’ long sequence of ERE, there was evidence that an even shorter stretch within could elicit R2 upregulation in our system. We defined a 52-long nucleotide sequence as the minimal region necessary and located two stretches of 18 and 16 nucleotides within this sequence that were essential but needed to be expressed in the context of the more extended sequence. Analyzing these essential nucleotides, we saw a strong homology with a sequence inside the R2 transcript. The homology sequence is located around the translation start site of R2 (RRM2), the ERE target gene, and we termed this homology region R2-box. Cells with insertion or deletion mutations (indels) in the genomic R2-box poorly expressed R2 and inefficiently supported HBV infection. Remarkably, these cells became ERE refractory. 

ERE induces the ATR-Chk1-E2F1 axis, the axis activated by UV. The finding that cells with indels in the R2-box are ERE irresponsive raised the question of whether these cells are UV irresponsive. Our data suggest that this is not the case and the mutated cells remained UV responsive. Based on these findings, we proposed a model whereby the region of R2 homology with ERE is exclusively important for R2 upregulation (Figure 4D). At this stage, we do not know how the homology region is involved. An attractive possibility is that ERE directly targets R2-box to generate a certain kind of hybrid, e.g., an R-loop. R-loops were reported to activate ATR [27,28,29]. Thus, the induction of the ATR-Chk1-E2F1 axis by ERE might be mediated via the R-loop. This interesting possibility has to be addressed in the future.

Different viruses have developed various strategies to upregulate R2 expression. Some large viruses (over 100 kb) encode their R2 gene, amongst them some herpes- and poxviruses [30,31]. Other DNA viruses dysregulate the cell cycle and push the cells towards S-phase and dNTPs synthesis [32,33,34,35]. Yet another group of DNA viruses induces RNR via the activation of DDR. A long list of viruses manipulates DDR for their benefit [36,37]. Additionally, HPV upregulates R2 expression by activating the ATR-Chk1-E2F1 pathway [38,39]. Epstein–Barr virus (EBV) and Kaposi’s sarcoma-associated herpesvirus (KSHV) also activate DDR [40,41]. In all the described cases, the RNR is induced by virus-encoded proteins. Here, we describe a novel mechanism whereby a small RNA fragment of HBV is sufficient to upregulate RNR. Whether other viruses have adopted similar mechanisms is an important open question.

## Data Availability

Not applicable.

## Supplementary Materials

The following supporting information can be downloaded at: https://www.mdpi.com/article/10.3390/cells11142248/s1, Figure S1: Schematic of HBV genome organization.; Figure S2: Structure predictions and RNA expression of ERE sequence analysis. Figure S3: Characterization of the R2 promoter and sequence similarity between ERE and R2 gene and the HepG2 R2 box knockout cell line. Figure S4: Establishment of HepG2 and HepG2 R2 mut cells that express NTCP and overexpress R2.
