# Supplementary material for: Evidence for a Hepatitis B Virus Short RNA Fragment Directly Targeting the Cellular RRM2 Gene
Source: Cells. 2022 Jul 20;11(14):2248. doi: 10.3390/cells11142248 (PMC9318981; doi:10.3390/cells11142248)
Supplement: Supplementary file 1 [file cells-11-02248-s001.zip › cells-1794284-supplementary.pdf]

Supplementary material

Evidence for a Hepatitis B Virus Short RNA Fragment Directly Targeting the Cellular RRM2 Gene

Karin Broennimann, Inna Ricardo-Lax, Julia Adler and Yosef Shaul

Table of contents:

Plasmid list.....…………………………………………………….….....................2

Primer sequences………………………………………………………….….…...........3

Antibodies…..…………………………………………………………………….......4

Supplementary figures

Figure S1..5

Figure S2..6

Figure S3..7

Figure S4..8

Plasmid List.

Plasmid name	Plasmid backbone	Location of insert	Insert
Empty vector	pLenti4 (Invitrogen)	Gateway LR sites	No insert
HBx (full length) and truncated/mutated versions	pLenti4 (Invitrogen)	Gateway LR sites	HA-tag 5′<br>HBx (nucleotides 1374-1838)<br>HBV 3′UTR (nucleotides 1838-1989 genotype A, adw2)
no X ORF	pLenti4 (Invitrogen)	Gateway LR sites	HA-tag 5′<br>HBV 3′UTR
HBV	pHR′ CMV GFP, Inder Verma lab, Addgene plasmid # 14858	XmaI - XhoI	1.3x HBV, 1.3x copies of the HBV adw2 strain from unique EcoRV site at its 5' end to the unique Taq1 site at its 3' end (position 1043 and 2017 relative to EcoRI restriction site)
pX330-U6-Chimeric_BB-CBh-hSpCas9	Feng Zhang lab, Addgene 42230		Guides were designed and inserted according to the Zhang lab protocol: https://www.addgene.org/crispr/zhang/
pU6-(BsaI)_CBh-Cas9-T2A-mCherry sgRNA for R2 start	pU6-(BsaI)_CBh-Cas9-T2A-mCherry<br>Addgene plasmid # 135012		sgRNA for R2 start site
pWPI hNTCP	pWPI-puro, Staphan Urban lab		
pcDNA3 RRM2	pcDNA3.1 (+), Edward Whang lab		

qRT-PCR Primers

R2 fw 5′AGAGAGTAGGCGAGTATCAGAGG

rev 5′CAAGTAAGGGCACATCTTCAGTTC,

18S ribosomal RNA fw 5′TCGGAACTGAGGCCATGATTAAG

rev 5′CGGAACTACGACGGTATCTGATC

HBx 3′UTR fw 5′CATGTCCCACTGTTCAAGCC

rev 5′TCTGACGGAAGGAAAGAAGTCA

HBx ORF fw 5′CCACCGTGAACGCCCATC

rev 5′TTGTGCCTACAGCCTCCTAATAC

HBs ORF fw 5′ACATCAGGATTCCTAGGACC

rev 5′TATCGCTGGATGTGTCTGCG

hNTCP fw 5′ TGCCTCAATGTTCTTCAGCC

rev 5′ TGTTCATGTTGTTCTTCATC

R2 overexpression fw 5′ TAGCCTTTGCTGCAGTGGAA

Rev 5′ CCAGGCATCAGTCCTCGTTT

Primers and probe for detection of intra- and extracellular HBV DNA:

5′-CCGTCTGTGCCTTCTCATCTG-3′ (sense),
5′-AGTCCAAGAGTCCTCTTATGTAAGACCTT-3′ (anti sense),
5-/56 FAM/CCGTGTGCA/ZEN/CTTCGCTTC ACCTCT GC/3IABkFQ/-3 (probe).

Primers for guides

Guide R2 start fw: caccgACGGAGGGAGAGCATAGTGG

Guide R2 start rv: aaacCCACTATGCTCTCCCTCCGTc

Antibodies

Rabbit anti-RRM2 (Cell Signaling E7Y9J Rabbit mAb #65939)

Mouse anti-Actin (Sigma A4700)

Mouse anti-Vinculin (Sigma)

Horseradish peroxidase–conjugated secondary antibody (Jackson)

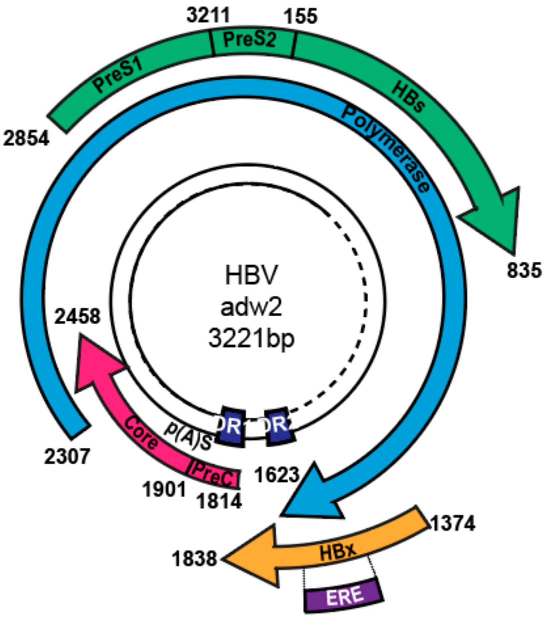

Figure S1. Schematic of HBV genome organization. Numbering starts from the EcoRI site. In addition to the main HBV ORFs we have depicted ERE within HBx.

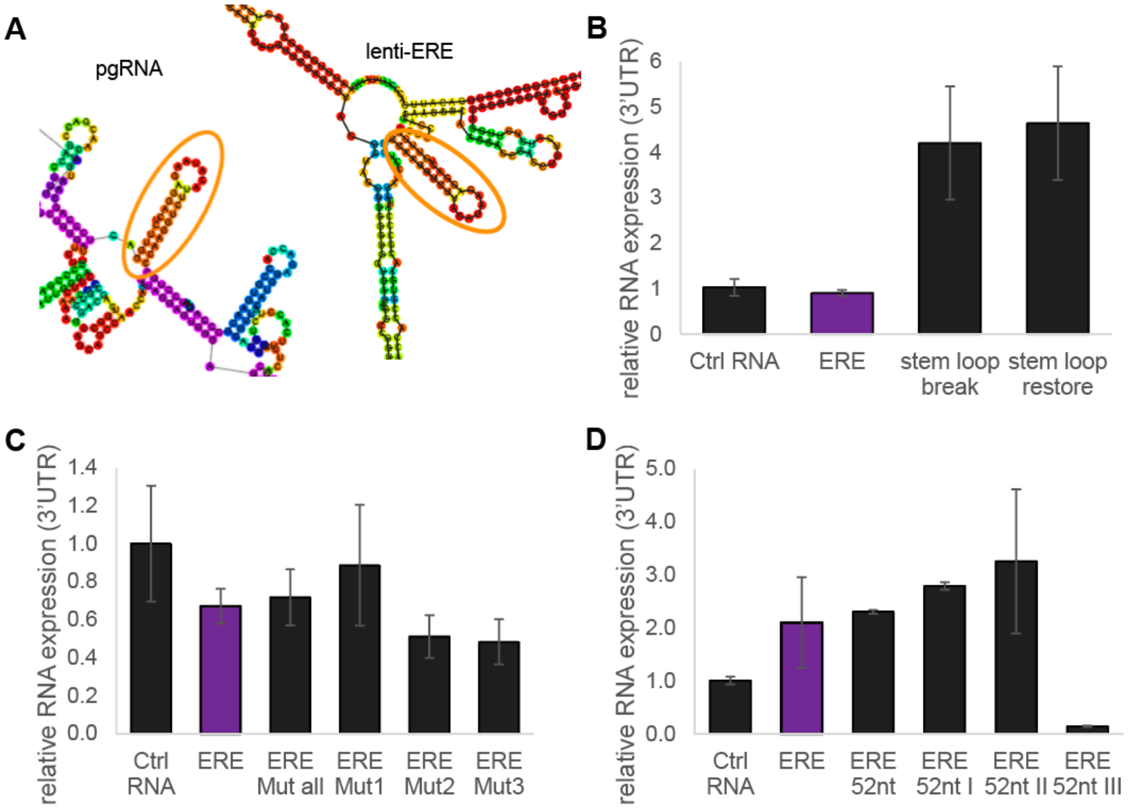

Figure S2. Structure predictions and RNA expression of ERE sequence analysis. (**A**) Structure predictions of ERE stem loop in context of HBV pgRNA and lenti-ERE sequence with RNA fold web server. (**B**–**D**) Quiescent HepG2 cells were transduced with lentivirus with the respective sequence depicted in Figure 1B. Cells were harvested three days after transduction and lentiviral expression level (3′UTR) of three biological replicates was analyzed by qRT-PCR.

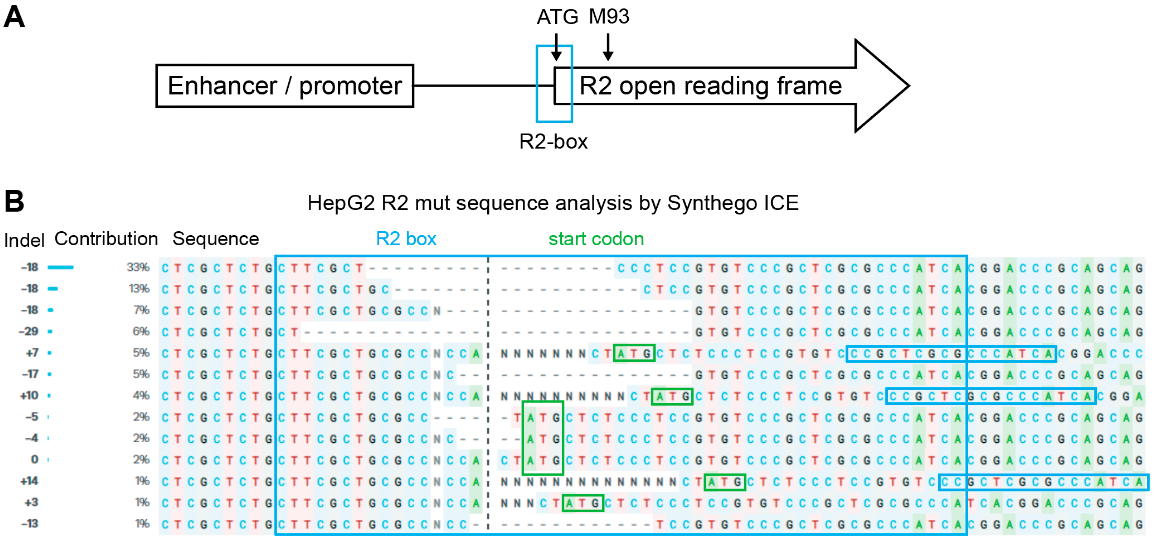

Figure S3. Characterization of the R2 promoter and sequence similarity between ERE and R2 gene and the HepG2 R2 box knockout cell line. (**A**) Schematic representation of the RRM2 (R2) locus with the R2-box in blue and the major ATG in and the downstream ATG at M93 indicated with an arrow. (**B**) Sequence analysis of R2 mut cells in the region around R2 translation start site by Synthego ICE web analysis tool (Synthego Performance Analysis, ICE Analysis. 2019. v2.0. Synthego).

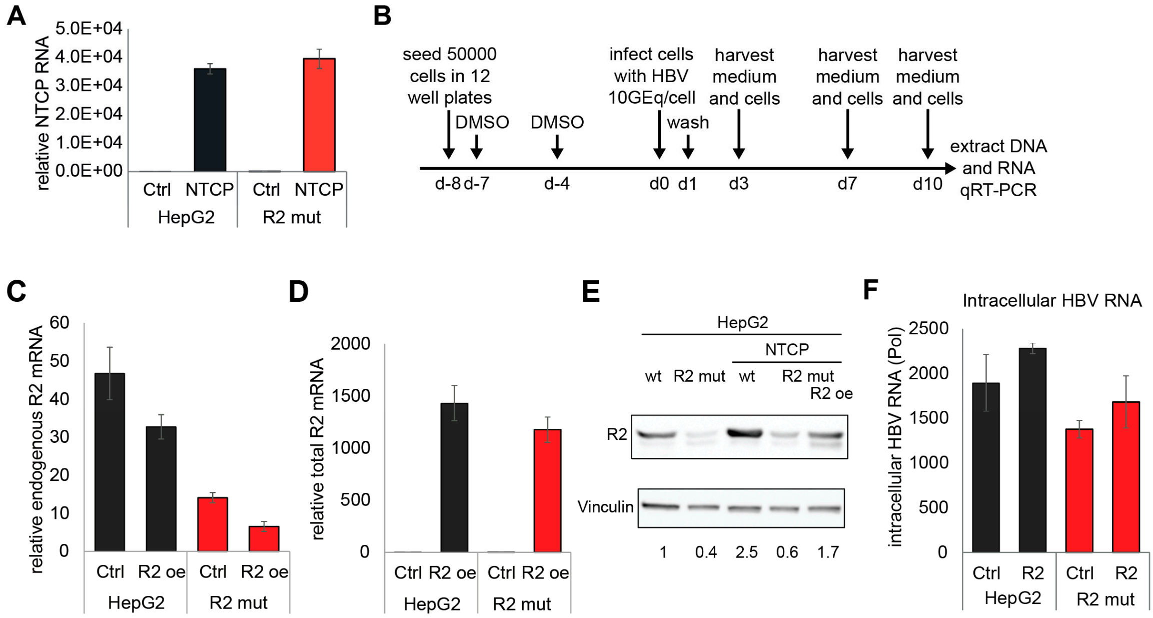

Figure S4. Establishment of HepG2 and HepG2 R2 mut cells that express NTCP and overexpress R2. (**A**) HepG2 and HepG2 R2 mutated cells that express NTCP and the original cell lines without NTCP were grown, RNA was extracted and NTCP expression was measured by qRT-PCR. (**B**) Schematic of experiment setup: Quiescent HepG2 and R2 mut cells expressing the NTCP receptor were infected with HBV (10 Genome Equivalents (GEq) per cell). Medium and cells were harvested on the indicated days post infection. DNA was extracted and qRT-PCR with HBV specific primers and probe was performed. (**C–E**) Stable cell lines expressing R2 from a CMV promoter were established. Quiescent cells were harvested and qRT-PCR with primers specific for the endogenous R2 only (**C**) or together with the ectopically expressed R2 (**D**) and WB of R2 were performed. (**F**) Experiment described in panel (**B**) was performed with the R2 overexpression cell lines and qRT-PCR of intracellular HBV RNA was performed.
